# A methodological framework for assessing development solutions: application to wood fuel challenges in Nigeria

**DOI:** 10.1038/s41598-025-97815-5

**Published:** 2025-04-16

**Authors:** Jamie A. Carr, Aliyu Salisu Barau, Eleanor K. K. Jew, Joshua D. Kirshner, Robert A. Marchant, Abubakar Tanimu Salisu, Gillian Petrokofsky, Julia Tomei, Lindsay C. Stringer

**Affiliations:** 1 Leverhulme Centre for Anthropocene Biodiversity, University of York, York, UK; 2 Department of Environment and Geography, University of York, York, UK; 3 York Environmental Sustainability Institute, University of York, York, UK; 4 Department of Urban and Regional Planning, Faculty of Earth and Environmental Sciences, Bayero University Kano, Kano, Nigeria; 5 Interdisciplinary Global Development Centre, University of York, York, UK; 6 Oxford Systematic Reviews, 266 Banbury Road, Oxford, UK; 7 Oxford Long-term Ecology Lab, Department of Biology, University of Oxford, South Parks Road, Oxford, UK; 8 UCL Institute for Sustainable Resources, University College London, London, UK

**Keywords:** Environmental impact, Energy policy, Energy security, Energy management, Risk factors

## Abstract

Development interventions often yield co-benefits and trade-offs across multiple Sustainable Development Goals (SDGs). However, current approaches typically assess progress towards specific SDG targets, such as increasing access to clean energy or improving health outcomes, rather than evaluating the co-benefits and trade-offs of the solutions used to achieve these targets. This study introduces a solutions-oriented methodology to assess the impacts of development solutions, applied to the case of wood fuel cooking-related challenges in Nigeria. Using a rapid evidence assessment and stakeholder workshop, we identify co-benefits, trade-offs, and barriers associated with 13 wood fuel-related solutions, classified into three types: enhancing fuelwood availability, adopting alternative technologies, and implementing external interventions. We find solutions that increase wood fuel availability can address environmental and social issues, but not health challenges, while alternative fuels/technologies face affordability, market, and cultural acceptance barriers. We highlight data limitations and propose an iterative process to comprehensively evaluate solutions’ impacts. This process facilitates context-specific, cross-sectoral planning but underscores that no universal solution exists. Successful interventions require multi-sector collaboration, public education, and strengthened governance to balance competing priorities and ensure equitable outcomes. By advancing solutions-based approaches, this study contributes to integrating SDG interactions into practical, evidence-informed policy and programming.

## Introduction

Identifying how multiple development challenges can be addressed simultaneously is increasingly necessary given the close interactions between the 17 United Nations Sustainable Development Goals (SDGs)^1^. Solutions (specific interventions, technologies, or practices designed to address development challenges), can create co-benefits (desirable outcomes) for other goals, as well as trade-offs (undesirable outcomes). Understanding these interactions can guide decision-makers to deliver more equitable and efficient solutions. Current approaches predominantly evaluate progress towards specific SDG targets rather than assessing the impacts of the solutions themselves, leaving a gap in understanding the details of how interventions operate in real-world contexts. This paper advances a solutions-oriented methodology to assess the interactions inherent in development solutions, explicitly integrating consideration of the co-benefits and trade-offs.

To date, efforts to document and map SDG interactions (the synergies (mutually reinforcing outcomes) and trade-offs (conflicting outcomes) that arise when progress is made towards one SDG relative to others) have tended to focus at the target level. Literature reviews, expert elicitation, correlative methods, and combinations of each^2–6^ have provided valuable insights into how progress toward one SDG target may influence others. However, these approaches often assume that interactions can be uniformly attributed to any intervention targeting a specific area of development, without considering how the choice of solution mediates these interactions. Breur et al.^7^ observe that while various approaches can identify SDG interdependencies, most lack practical applicability for policy or planning. A growing body of work highlights the need to move beyond target-oriented assessments to focus on solutions themselves^8,9^, recognising that interventions seeking the same outcome may yield different co-benefits and trade-offs depending on their design, implementation, and context^10,11^. However, developing new methodologies to address these shortcomings remains nascent and requires further attention.

Target-oriented approaches assess SDG interactions by examining progress towards specific SDG targets, often measured through aggregate indicators. In contrast, solutions-oriented approaches focus on the practical interventions used to achieve these targets. While target-oriented assessments are valuable for monitoring global progress, they fail to capture the complexities of implementation, particularly in contexts where interventions intersect with socio-economic, cultural, and environmental factors. Shifting to a solutions-oriented perspective, this paper aims to provide decision-makers with a more nuanced approach, enhancing understanding of how interventions operate in specific contexts, enabling them to maximise synergies and mitigate trade-offs.

This paper takes the cross-cutting development challenge of wood fuel use for cooking as a case to develop and test a new solutions-oriented approach. Wood fuel and charcoal (hereafter wood fuel), provide a critical energy source for millions in the global south, and usefully exemplify the interconnected challenges posed by development interventions. Wood fuel use links directly to SDG 7 (affordable and clean energy)^2,12,13^, and to multiple other SDGs. Shifting away from wood fuel energy sources has proved notoriously difficult^14–16^ yet is vital to achieve multiple SDGs. Against this background, we introduce a methodology for assessing the interactions of development solutions with SDG targets and apply it to the specific example of wood fuel use in Nigeria to test and illustrate its utility.

Rather than merely recognising challenges, our solutions-oriented approach focuses on the specific interventions, technologies or practices expected to address a problem, making it invaluable for identifying the context-specific barriers to particular interventions^17^. By building in stakeholder engagement across multiple sectors, this approach can help anticipate implementation obstacles across social, cultural, economic, political, and practical dimensions^18^ and foster a comprehensive understanding of the intervention landscape and its context-specific complexity. Moreover, it identifies potential trade-offs and co-benefits^19,20^, offering insights into the nuanced impacts of interventions. These considerations are crucial to guide responsible development interventions, based on project plans that reflect complex realities and cross-sectoral decision-making, and balancing competing priorities.

Our empirical analysis focuses on Nigeria, which, like many sub-Saharan African countries, relies heavily on wood fuel for cooking energy due to large energy supply gaps and costs of alternatives. Due to this demand and a large population, Nigeria experiences high levels of forest loss/degradation; for example, between 2001 and 2023 Nigeria lost an estimated 13% of its tree cover^21^, with wood fuel a key driver^22^. The country faces high poverty levels and multiple inequalities (e.g. economic, gender), particularly in rural areas where infrastructure is poor and access to basic facilities like healthcare and education is limited^23^. The population’s dependence on small-scale agriculture and natural resource extraction leaves them vulnerable to shocks, including from climate change-related events. Nigeria also struggles with high crime rates, corruption, ineffective governance, and regional conflicts and insecurities^24^. It is culturally diverse, with over 500 local languages, and has a large, dense, and rapidly growing population, currently estimated at over 227 million and projected to become the world’s third most populous country by 2050^25^. Nigeria’s significant oil and gas resources, although mostly exported, offer economic and energy opportunities domestically. These factors must be considered when developing holistic, cross-sectoral development strategies, including those supporting affordable and clean energy and the transition away from wood fuel. Specifically, this paper asks: (1) What are the co-benefits and trade-offs for solutions to wood fuel-related challenges in Nigeria?; (2) What are the major barriers to implementing these solutions?; and (3) How can these findings guide decision-making to achieve equitable and sustainable outcomes?

Against this background, we use a rapid evidence assessment (REA) to synthesise the available evidence, verifying and supplementing it through consultations with relevant stakeholders and experts in a workshop. We provide an overview and comparison of the co-benefits, trade-offs and barriers associated with each potential solution and outline a process that can support decision-making.

## Results and discussion

**Fig. 1:**
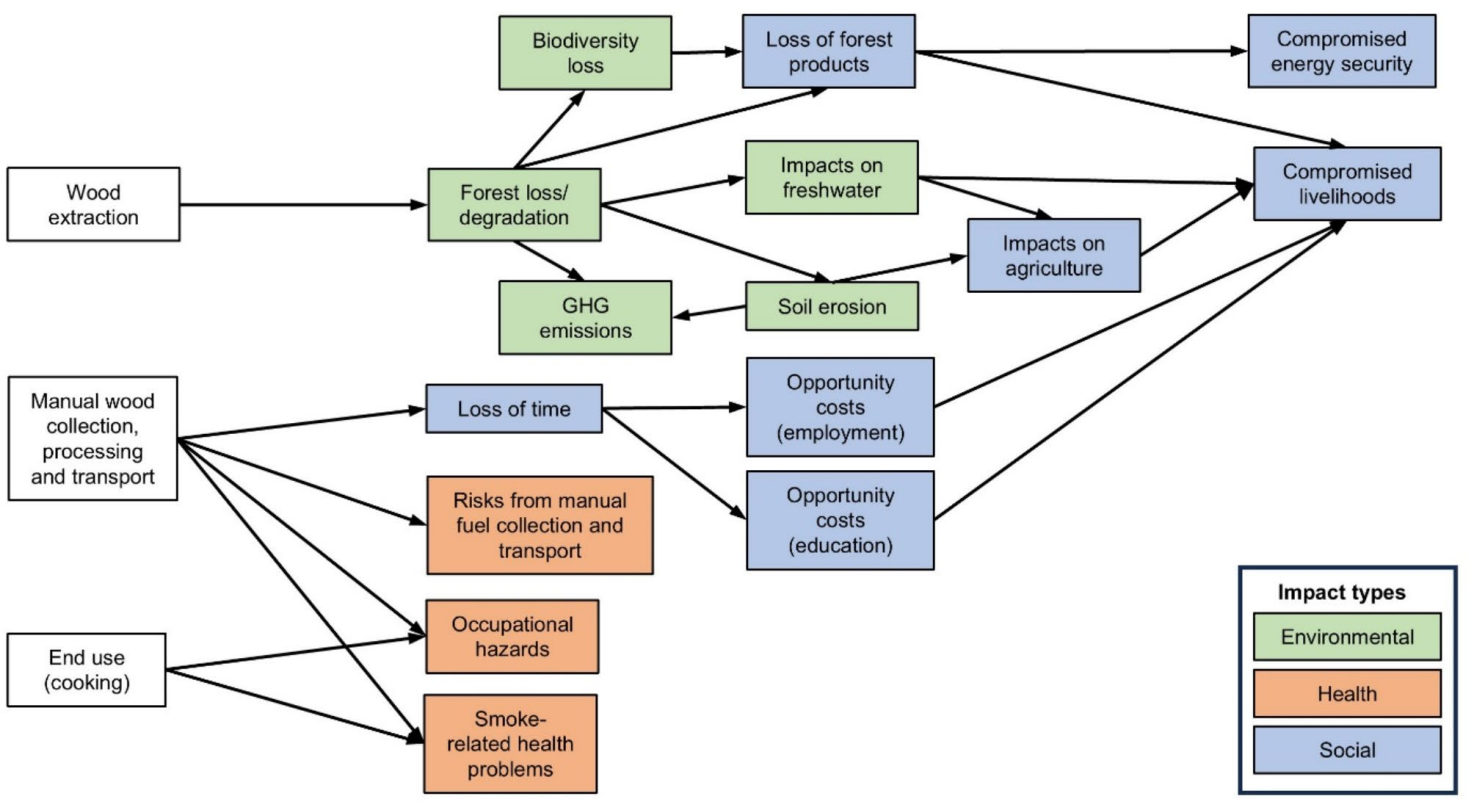
Direct and indirect negative impacts associated with the collection, processing, transport and use of wood fuel for cooking in Nigeria.

Our REA yielded 91 documents meeting our predefined inclusion criteria, which were later supplemented by information gathered at a stakeholder workshop (see “Methods”). The REA documented 15 categories of wood fuel-associated challenges in Nigeria, which we classified as either environmental, social, or health-related (Fig. 1). These challenges highlight the complex and interlinked nature of wood fuel use within development contexts and its interaction with multiple SDGs.

Wood fuel harvesting directly contributes to loss/degradation of forests and other tree-dominated habitats^26–28^ (hereafter ‘forests’), leading to biodiversity loss^28–30^ and soil erosion^27,31^ (SDG 15), emitting greenhouse gases that contribute to climate change^32^ (SDG 13) and threatening freshwater systems and the services they provide^31,33^ (SDG 6). Compromised long-term sustainability of energy supply^26,34^ (SDG 7), reduced agricultural productivity and viability^30,34^ (SDG 2) and loss of natural products upon which many people depend for subsistence and income^34^ can further jeopardise people’s well-being and livelihoods (SDG 1). Beyond economic and subsistence impacts, compromised energy security also affects household well-being by limiting access to essential energy-dependent services such as room heating, safe drinking water, and warm water for hygiene. The significant time spent collecting wood fuel (typically by women and girls) presents opportunity costs that prevent them from pursuing other livelihood-supporting activities, including formal employment or education^30,35^ (SDGs 4 and 5). Wood fuel collection, processing and usage has significant health impacts in both domestic and commercial contexts, disproportionately affecting women and children (SDGs 3, 5 and 8). Manual transport of heavy wood fuel loads (often from increasingly distant locations) can cause fatigue and spinal problems^30^, while preparing fuels for sale (especially charcoal) has a range of associated health problems (e.g. cuts, injuries and respiratory problems)^36,37^ (SDGs 3 and 8). Cooking with wood fuel exposes end-users in both domestic and commercial settings to harmful smoke and particulates, causing a range of cardiovascular and other conditions (e.g. eye problems)^38–40^, and potentially increasing child mortality^41,42^ (SDG 3).

### Proposed solutions and their effectiveness

The REA also revealed 13 solutions that have been shown, or might otherwise be expected, to help address one or more of these challenges. Identified solutions fall into three main types (Table 1): ‘Type 1’ solutions seek to maintain or enhance wood fuel availability, ‘Type 2’ solutions involve changes to cooking technologies or practices, and ‘Type 3’ solutions comprise interventions (typically by an external agency) to facilitate or enforce a change in practice or behaviour. Although Type 3 solutions are predominantly intended to facilitate outcomes via solutions from the other two groups, they can each have associated barriers, trade-offs and co-benefits that require consideration, so are included as standalone solutions.

**Table 1:** Types of solutions encountered in the literature, including working definitions used in this paper.

Solution type	Solutions	Definitions
Type 1: Fuel wood supply	Afforestation / reforestation (Forestation)	Intentional creation of new/restoration of lost or degraded areas of forest (or similar wooded habitats) using native species. This can include the direct replacement of trees shortly after they are felled.
	Agroforestry	The incorporation of trees (native, exotic or mixed) into agricultural areas and practices.
	Shelterbelts/windbreaks	A line of trees or shrubs (native, exotic or mixed) planted to protect an area from fierce weather and/or erosion.
	Woodlots/plantations	Purposefully planted stands of trees (native, exotic or mixed) on private or community owned land to provide wood fuel (among possible other uses). Wood grown may be used directly and/or sold.
	Participatory forest management (PFM)	Management of forests (or other wooded habitats) by local communities and/or other relevant stakeholders to ensure that offtake is sustainable and fair.
Type 2: Technology change	Non-renewable fuels and technologies	Non-wood cooking fuels (and appropriate stoves) that are derived from non-renewable (typically fossil-based) sources, including kerosene, natural gas and LPG,. Not including electric stoves.
	Renewable biomass fuels	Non-wood cooking fuels (and appropriate stoves) that are derived from renewable sources such as agricultural residues, and animal/human waste, including biogas, ethanol, and briquettes.
	Electric stoves	Cooking stoves that run solely from an electricity source, rather than from direct burning of a fuel source to produce cooking heat. Electricity may be sourced from either renewable or non-renewable sources.
	Improved cookstoves (ICS) for wood-based cooking	Wood-/charcoal-burning cookstoves with improved designs that increase burning efficiency and/or reduce emissions.
	Improved ventilation	Increasing ventilation (e.g. using chimneys, windows or relocating outside) in a home or workplace to reduce excessive exposure to smoke and airborne particulates.
Type 3: External interventions	Livelihood-related interventions	Provision of alternatives, compensation, or incentives (in either monetary or non-monetary form), to reduce the prevalence of damaging/undesirable activities.
	Legal/regulatory interventions	Action on the part of the government, police or other authorities to develop and enforce legal or regulatory changes that prohibit/limit one or more activities.
	Education/awareness-raising for behaviour change	Targeted education or awareness-raising designed to encourage the use of one practice over another.
	Improved access to health services/equipment	Any action that allows people greater access to health services (e.g. building hospitals, providing insurance) or to health and safety equipment.

**Fig. 2:**
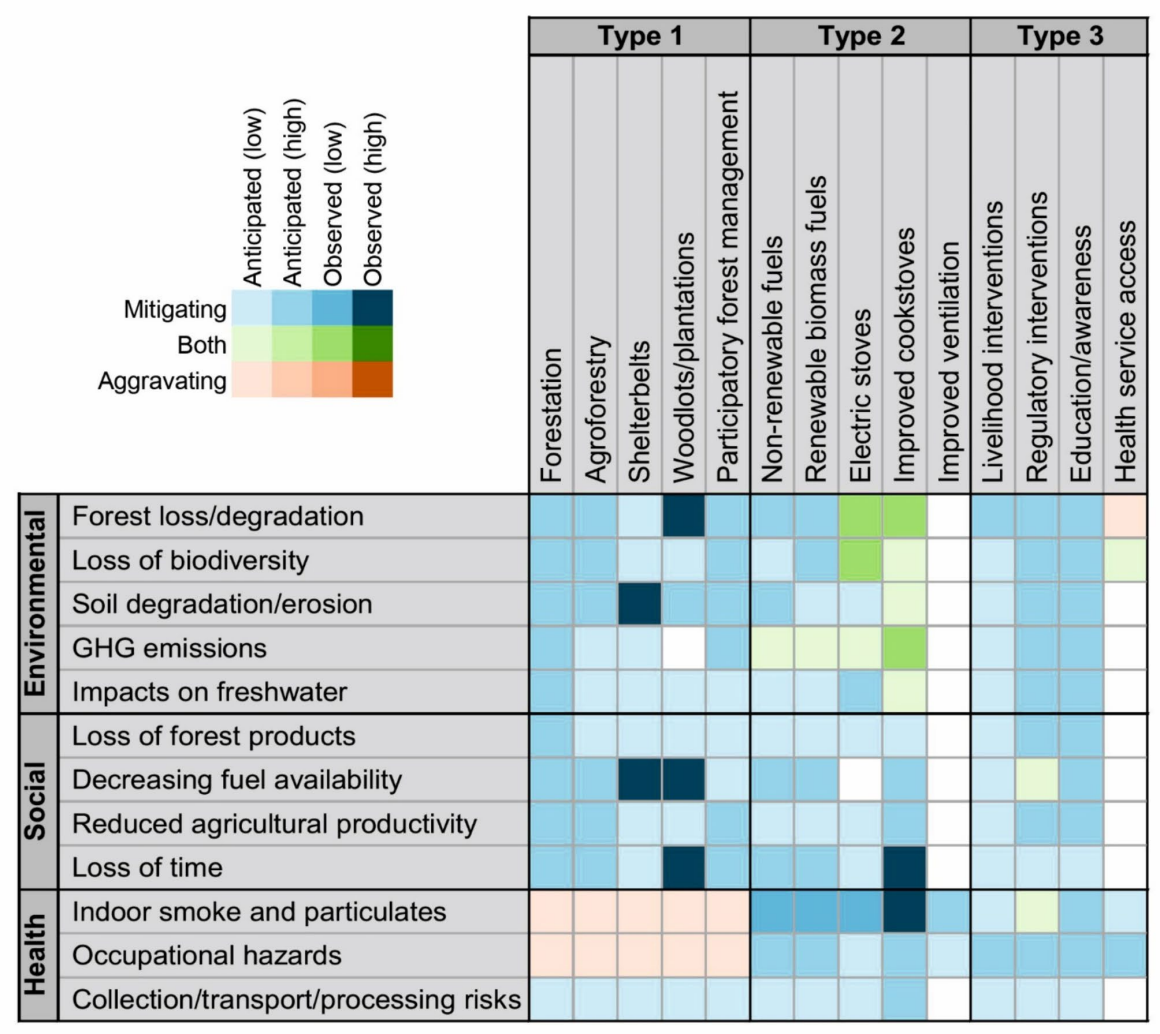
Wood fuel-related impacts and the solutions that are expected to mitigate them, aggravate them, or both. Colour scheme shows the strength of evidence associated with each impact-solution combination (see “Methods” for details).

Figure 2 summarises all recorded solutions from the REA, including the specific impacts that they might be expected to address and the associated strength of the evidence. Although a large proportion of the 143 impact-solution combinations have expected outcomes (whether positive or negative), only 14 (9.5%) are based on empirical observations, while the remainder are based on expected potentials alone. This highlights an urgent need to increase the evidence base around the effectiveness of options to deliver expected benefits. Such insights will improve the effectiveness of investments in addressing priority goals.

Some Type 1 solutions (e.g. woodlots and plantations) fall short in offering clean energy (SDG 7) and climate action (SDG 13) yet can largely address all other wood fuel-associated environmental and social impacts. Afforestation/reforestation (hereafter ‘forestation’) outcomes are uniformly anticipated both in the literature and by workshop participants. Despite not featuring in our evidence base from Nigeria, logic suggests that with sufficient effort and appropriate techniques (e.g. the correct choice of species) replanting native forests would help to enhance many of their associated services. Participatory forest management (PFM) can accompany all other Type 1 solutions to enable knowledge and expertise to be shared on best practices (e.g. species selection, planting techniques), and, in the case of forestation and shelterbelt establishment (typically on non-private lands), to ensure offtake remains equitable and sustainable. Consultations also suggested that information provision through PFM can have wider benefits for public education.

Woodlots/plantations, agroforestry plots and shelterbelts generally provide alternative wood sources rather than directly influencing the size or structure of natural forests. This needs to be understood in the context of the anticipated rapid human population increases in Nigeria and associated high deforestation rates. It remains unclear to what extent these alternative sources can provide the volumes of wood fuel required while still performing their primary functions. Given these systems may have limited capacity to deliver certain benefits (e.g. those relating to biodiversity^43^ and carbon sequestration^44^) compared with natural forests, we encourage accompanying efforts to restore and protect natural forests, rather than treating these as standalone solutions.

Type 1 solutions are largely expected to deliver subsistence and livelihood-related co-benefits through provision of usable/saleable products. Although woodlots may be cultivated with the sole purpose of providing wood fuel^45^, in the case of agroforestry plots and shelterbelts, the potential for a conflict of purposes exists, wherein extraction of fuel from these sources undermines agricultural and soil protection benefits, respectively, or vice versa. Observations from Kano State showed that farmers became disappointed and resentful following establishment of shelterbelts, when the anticipated financial benefits were not forthcoming^46^. Tree planting can also present a trade-off with space for other potential uses (e.g. agriculture), and, despite providing benefits in terms of soil retention, may negatively impact agriculture as roots encroach into cropped areas^46,47^.

Beyond mitigating risks associated with manual fuel transport (assuming more trees means less distance to travel for fuel), Type 1 solutions have limited ability to address wood fuel-related health impacts. Workshop participants were concerned that increased availability of wood fuel may inadvertently increase usage, thereby increasing instances of both occupational health hazards and those arising from indoor domestic cooking. Nevertheless, some non-wood fuel-related health benefits may arise from Type 1 solutions (e.g. reducing heat exposure through shade provision and microclimate regulation, and through provision of medicinal plants).

Type 2 solutions include improving ventilation (via chimney installation, windows, or relocating cooking outdoors) to mitigate smoke-related health problems. However, this solution does not address other wood fuel challenges. More comprehensive Type 2 solutions involve alternative cooking fuels and technologies, particularly electric stoves and renewable biomass technologies, which have the potential to tackle multiple wood fuel-related issues and offer significant co-benefits. Electric stoves, for instance, necessitate expanding reliable electricity access, which can enhance the economy, education, and overall well-being^48,49^. Utilising waste products for energy, such as in renewable biomass technologies, can improve hygiene in areas with poor waste management and produce agricultural fertilizers as byproducts^48,49^. Both solutions can reduce GHG emissions from forest loss and degradation if appropriately implemented. For electric stoves, this means using renewable energy sources instead of fossil fuels. For renewable biomass fuels, such as biogas, it requires technologies that remove CO_2_ and impurities^50,51^. However, expanding infrastructure for grid-connected electricity may have trade-offs for natural ecosystems^52,53^, making off-grid renewable systems preferable. Similarly, renewable biomass fuels could compete with natural habitats and food production if site selection for feedstock production is poorly considered^54,55^. Therefore, using more readily available feedstocks derived from human, animal, or agricultural waste is recommended to minimise these trade-offs.

Technologies that derive energy from non-renewable fuels (e.g. liquefied petroleum gas (LPG), kerosene and natural gas) have similar capacity to address social and health-related impacts as those that use renewable biomass but have limited capacities to reduce GHG emissions. Although these fuels may reduce emissions from forest loss and degradation, they remain fossil fuels, so emissions may be shifted to another source rather than eliminated. Although work has demonstrated these technologies to be cleaner than wood fuel, this may only be in a relative sense, and some fuel types (notably kerosene) can still have undesirable health impacts^56,57^.

Improved cookstoves (ICS), designed to burn wood fuel more cleanly and efficiently, were the most frequently suggested/investigated alternative cooking technology in our REA. ICS have been associated with positive outcomes for forest loss, GHG emissions, loss of time and smoke-related health hazards^32,58–60^ and can be expected to mitigate all other recorded impacts too. However, it was widely agreed in workshop consultations that widespread promotion of ICS could ultimately increase dependence on wood fuel, and despite reducing environmental impacts, may not eradicate them completely, particularly given Nigeria’s projected population growth. Furthermore, some studies have demonstrated a limited capacity for ICS to reduce smoke-related health impacts^35^, suggesting further developments in stove design are needed if this solution is to be adopted more widely.

Alternative cooking technologies present a double-edged sword in terms of impacts on livelihoods. While each alternative offers potential to generate new livelihood opportunities (e.g. from the manufacturing and installation of new technologies and infrastructure, or the production, distribution or sale of fuels^47,61^), groups currently making their living from the wood fuel sector should not be inadvertently left without a source of income. This is particularly relevant to those engaged in the collection/production of wood fuel, who are often among the poorest and most marginalised members of society^62,63^ and may lack the necessary skills and resources to transition to an alternative means of generating income.

Type 3 solutions vary in their respective abilities to address wood fuel-related challenges. Among these, efforts to improve education/raise awareness are relevant to all impacts, with seemingly no trade-offs (wood fuel-related or otherwise). Nevertheless, this may not be especially effective as a standalone solution and needs to be integrated with Type 1 and 2 solutions. The following section shows how low public awareness is a key barrier to be overcome for many other solutions to be effective.

Improving access to health services and equipment can mitigate challenges relating to indoor smoke-related risks and occupational hazards but has limited capacity to address environmental and social challenges. Expanding health-related infrastructure may have some negative impacts on natural systems but could help to mitigate the overexploitation of wild medicinal species (some of which are threatened by this process in Nigeria^64^) as people switch to more modern practices. Workshop participants also noted the key role that health services can play in education and awareness raising, including around alternative cooking fuels and technologies. Other health-related co-benefits of this solution are profound, given the currently poor status and accessibility of health services in Nigeria. Legislation and enforcement should ensure that employers provide adequate health and safety equipment in the workplace, whether concerning wood fuel or otherwise, as this would appear to be a no-regrets option.

Livelihood-focused interventions, which provide alternatives, compensation, or incentives (monetary or non-monetary), to reduce the prevalence of damaging or otherwise undesirable activities^65^, are expected to contribute to addressing all challenges. Mitigating forest loss also further mitigates subsequent environmental impacts, and some social and health impacts (manual fuel collection/transport risks, impacts on agriculture and loss of time). Livelihood-focused interventions can also help reduce both smoke-related health risks and occupational hazards by subsidising/incentivising alternative cooking technologies and forest-based livelihoods (e.g. beekeeping), respectively^29,66^.

Legal/regulatory interventions, which prohibit/limit one or more activities can be expected to facilitate mitigation of all impacts but may have unintended trade-offs for fuel availability if offtake is banned or highly restricted. Research conducted beyond Nigeria has shown that restricting offtake from forests can have negative impacts on people’s livelihoods, often affecting those on the lowest incomes^67^. Such interventions, especially when developed and enforced in a top-down manner, may inadvertently incentivise corruption, black markets and shadow economies (if demand remains while prices increase)^68^, which can lead to increased deforestation^69^.

## Identifying and overcoming key barriers

**Fig. 3:**
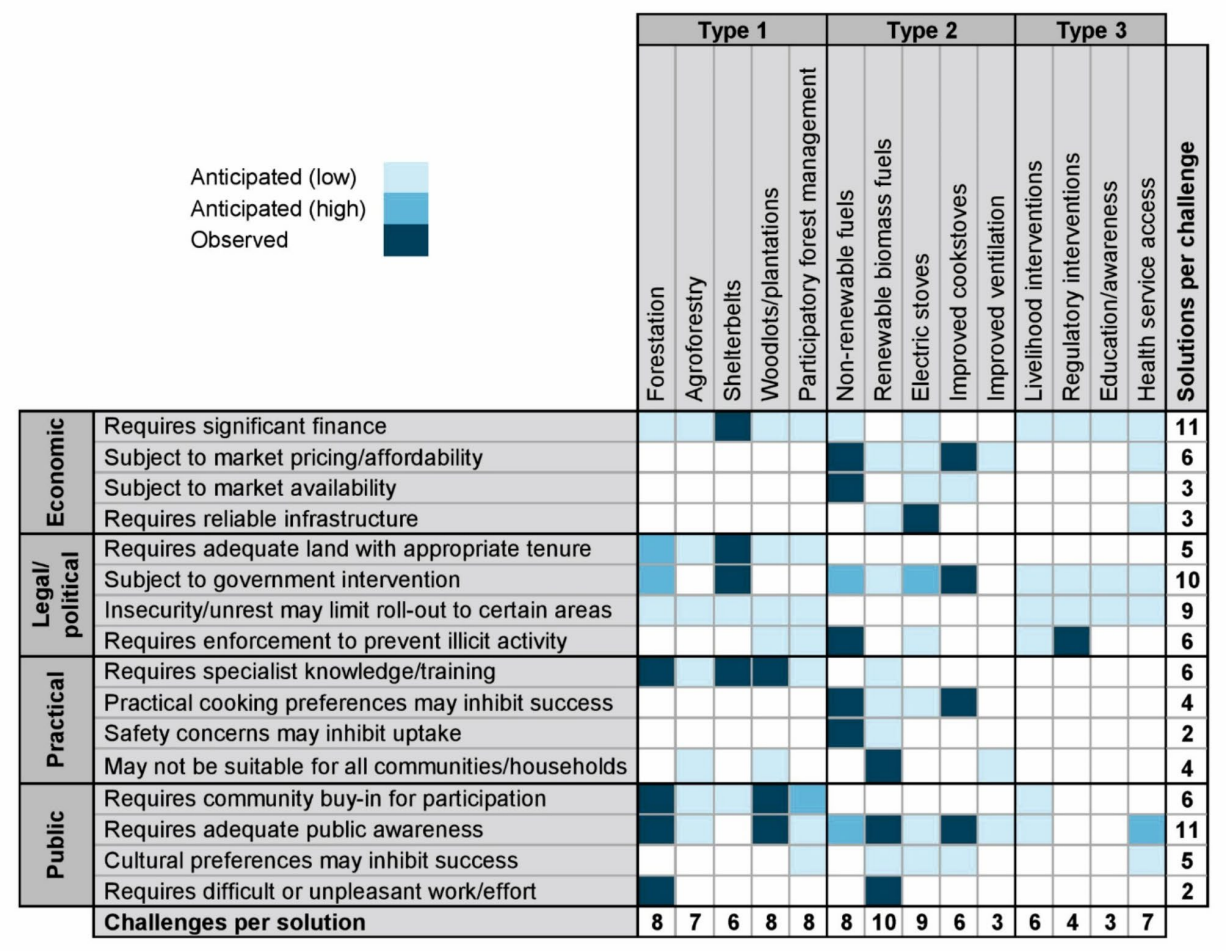
Recorded solutions to wood fuel-related impacts and the barriers that each might be expected to experience. Colour scheme shows the strength of evidence associated with each solution-barrier combination (see “Methods” for details).

We identified 16 major barriers that can hinder the implementation of solutions or their effectiveness in achieving their intended outcomes. These can be grouped into four main categories (Fig. 3). ‘Economic’ barriers relate to finance or markets and require some form of progress or stability in these areas for solutions to be successful. ‘Legal/political’ barriers relate to political bodies, institutions and/or the laws, policies, regulations and other instruments that they embody, as well as their implementation and enforcement. ‘Practical’ barriers relate to the ability for members of the public to implement the solution, while ‘public’ barriers relate to their willingness to do so.

Economic barriers for all Type 1 and Type 3 solutions include a requirement for significant financial investment from the government or other actors, also relevant to selected Type 2 solutions, but less so for renewable biomass fuels, ICS, and improved ventilation. Other economic barriers are most relevant to Type 2 solutions and increasing access to health services. Market pricing and affordability affect all Type 2 solutions. Evidence suggests that even subsidised costs of switching to alternative cooking fuels or technologies are prohibitive for some households^70^. Similarly, the inconsistent availability of alternative cooking fuels limits their uptake, leading to a reversion to wood fuel, except for renewable biomass fuels, which are generally expected to be consistently available. The need for widespread and reliable infrastructure, such as tarred roads and power grid connections, is a barrier to the roll-out of electric stoves and improved access to health services. Although some progress has been made, it remains slow due to insufficient, uneven and unreliable infrastructure^71,72^.

Legal/political barriers include a requirement for government intervention (itself reliant on relative political stability, will and capacity), and is among the most common barriers, relevant to 11 solutions. Only agroforestry, woodlots, PFM and improved ventilation, can proceed without such public intervention or significant external support. Other legal, institutional and political barriers include a requirement for adequate land with appropriate tenure security (relevant to all Type 1 solutions only), and for effective enforcement to prevent illicit activities (relevant to some solutions from each Type). Although not encountered in the literature, workshop participants felt that ongoing civil unrest may limit roll-out of Type 1 and 3 solutions in some areas.

Practical barriers include a requirement for specialist knowledge/training (e.g. on species selection and planting/cultivating techniques^26,46,47,73^), which applies to all Type 1 solutions. Other practical barriers are that alternative fuels/technologies may be unable to cook the style/quantities of food preferred by many Nigerians, while non-renewable and renewable biomass fuels are sometimes perceived as unsafe. A further challenge for some Type 1 and 2 solutions is that they may not be suitable for implementation by some households/communities. Agroforestry and woodlots typically require sufficient privately-owned land, renewable biomass fuels require sufficient locally available raw input materials, while improved ventilation may simply be impractical for some housing designs. Notably, no practical barriers are associated with Type 3 solutions.

Among public barriers, the need for public awareness and subsequent willingness for change is most frequently noted, equalled only by substantial financial requirements. Numerous documented examples highlight how low public awareness leads to poor engagement or uptake of solutions^26,47,58^, emphasising the importance of education and awareness-raising, which we consider as a standalone solution. Some Type 1 solutions require community-level buy-in, and livelihood-focused interventions may fail if insufficient people are engaged or if the scheme is viewed unfavourably. Cultural factors also pose barriers, such as attachment to traditional techniques related to forest management, cooking, and healthcare. For forestation and renewable biomass fuels, the often-arduous labour required has been shown to inhibit uptake^47,74^.

**Fig. 4:**
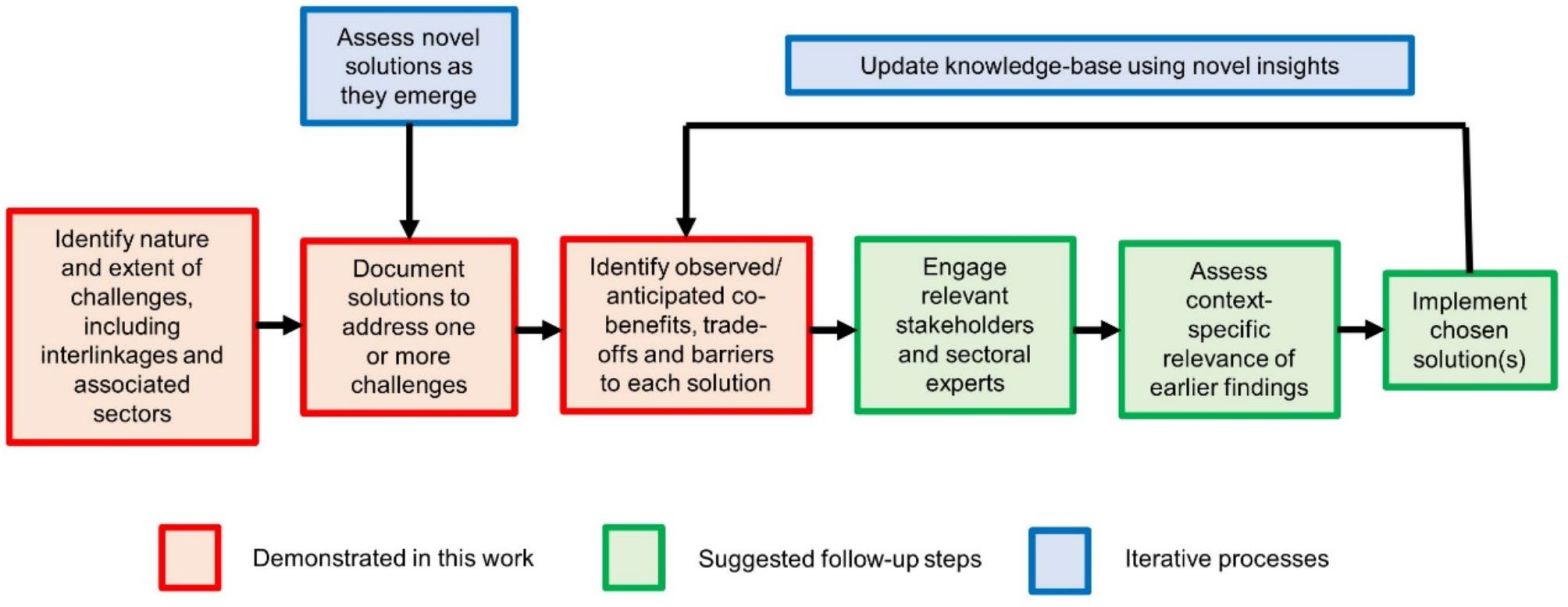
Steps in the process to guide decision-makers in selecting solutions, enabling a more comprehensive assessment of their potential effects and iterative development of the knowledge base.

We have demonstrated a structured approach to assess and compare the pros, cons, and barriers associated with various solutions to a cross-cutting development issue. While assessing such interactions is inherently complex, our methodology provides a systematic framework that helps decision-makers navigate these challenges in a transparent and comparative manner. Resulting information can provide valuable insights to inform project plans and subsequent interventions, helping to identify potential barriers, benefits and trade-offs across multiple SDGs. Given the limitations in available data, particularly the reliance on perceived and anticipated outcomes in some cases, these findings should be used as indicative rather than definitive, and are best complemented with additional empirical research wherever possible. Before implementing preferred solutions, participatory processes with local stakeholders should explore the relevance of potential trade-offs and co-benefits and establish the importance of identified barriers (Fig. 4), which was not attempted in this work. The approach should be iteratively updated with emerging insights and novel solutions. While we focused on wood fuel used in cooking, the approach is applicable to other multifaceted development challenges like clean water/sanitation or food security/agricultural sustainability, which involve complex, interlinked technological, behavioural, and political solutions.

In the near term, wood fuel use is likely to persist across much of Nigeria, necessitating urgent promotion of Type 1 solutions to overcome environmental and social challenges. Care must be taken to ensure that expanding Type 1 solutions doesn’t result in overreliance on their associated livelihood benefits, as this could hinder roll-out of Type 2 solutions and/or have negative impacts as these become increasingly less viable. Tree planting should be supported by capacity building for success and biodiversity benefits, and, if on common lands, by PFM to ensure long-term sustainability and equitable sharing of products and profits. Longer-term solutions to wood fuel-related challenges require a transition to alternative cooking fuels/technologies. Renewable biomass and electric stoves are the most promising options, addressing challenges with minimal trade-offs, provided environmental impacts and inequalities are managed. Despite this potential, these stoves face sizeable barriers, suggesting that widespread adoption will be challenging, even if they are co-designed with users. A key barrier is ensuring these stoves can cook comparable quantities and styles of food to traditional methods.

Nigerian national policies on wood fuel energy, such as the Energy Transition Plan^75^, National Energy Masterplan^76^, and National Clean Cooking Policy^77^, aim to promote alternative cooking methods, primarily focusing on LPG and ICS technologies, as well as establishing plantations of fast-growing tree species. However, past efforts to introduce these technologies and reforest have largely failed^78–80^, highlighting the need to adequately address the barriers and establish appropriate enabling conditions. While these policies note key co-benefits, such as livelihood opportunities from new technologies, they often overlook potential trade-offs, like loss of livelihoods for those in the wood fuel trade, GHG emissions from increased LPG usage, and environmental impacts from plantation expansion and infrastructure deployment. Prioritising an enabling environment and minimising trade-offs should be achieved through cross-sectoral dialogues and stakeholder consultations.

To successfully transition away from wood fuel, the Nigerian government and other agencies should aggressively pursue poverty reduction schemes, promote widespread education and awareness campaigns, and improve health services and their accessibility. These are no-regrets actions with notable co-benefits and few barriers beyond those intrinsic to the government. Regulatory measures should be deprioritised unless developed through bottom-up, participatory processes like PFM, as they may have unintended trade-offs and limited effect due to poor enforcement. Development agencies and NGOs should incorporate enabling conditions into their interventions, such as combining access to alternative cooking technologies with awareness campaigns and livelihood enhancements. Although some barriers, like those from civil unrest, are challenging to address, anticipating them can help develop strategies to mitigate their impacts on desired outcomes.

## Conclusions and recommendations

This study presents a methodology for assessing the impacts of development solutions, illustrated through the example of wood fuel-related challenges in Nigeria. The methodology’s solutions-oriented approach offers a practical framework for understanding the co-benefits, trade-offs, and barriers associated with interventions, contributing to the integration of SDG interactions into decision-making.

We have shown that wood fuel-related challenges are multifaceted, and span environmental, social, and health dimensions, requiring cross-sectoral solutions to balance competing priorities. We also show that no single solution can address all challenges; enhancing wood fuel availability addresses environmental and social issues but may exacerbate health-related impacts. Alternative technologies offer significant potential to reduce environmental and health risks but face affordability, cultural, and infrastructural barriers. Barriers are context-specific, and economic, legal, practical, and public barriers were found to significantly influence the feasibility and scalability of solutions, particularly in resource-constrained settings like Nigeria. Our work has also shown that notable evidence gaps persist. Most findings rely on anticipated or perceived impacts rather than direct empirical evidence, highlighting the need for further research and validation. While the methodology provides a valuable tool for decision-makers, its effectiveness depends on addressing these challenges and refining the evidence base to support sustainable and equitable outcomes.

To improve the impact and scalability of interventions, we make several recommendations. Strengthening participatory processes is crucial, with local communities and marginalised groups actively involved in decision-making to ensure solutions are equitable, culturally acceptable, and practical. Expanding stakeholder engagement to include end-users, particularly women and rural households, can enhance the relevance and uptake of interventions. Addressing systemic challenges is also vital; targeted investments in infrastructure, such as reliable electricity supply and improved transport networks, would support the adoption of alternative technologies, while financial incentives like subsidies or microloans could improve affordability for marginalised households.

Improving the evidence base is another critical priority. Conducting empirical studies and pilot projects to validate anticipated impacts and measure long-term outcomes would help close evidence gaps. Clearer criteria for assessing causation and evidence strength are also needed to enhance the reliability of findings. Integrated approaches that combine interventions across solution types, such as coupling alternative cooking technologies with education campaigns or livelihood support, are likely to yield more durable outcomes in the long term. Collaborative efforts between sectors, including energy, health, and environment, can harness more co-benefits and reduce trade-offs, fostering more holistic solutions.

Finally, enabling conditions must be enhanced through robust governance mechanisms, equitable resource management, and poverty reduction programmes. Policymakers and practitioners should prioritise initiatives that address underlying socio-economic inequalities, ensuring that interventions are both sustainable and equitable.

By implementing these recommendations, policymakers and practitioners can enhance the effectiveness of wood fuel-related interventions while contributing to broader SDG achievements. Iterative refinement of the methodology and its application across diverse contexts will further support sustainable development efforts globally.

## Methods

### Rapid evidence assessment

To derive our initial set of potential publications for inclusion, we searched three academic databases (CABI, Scopus, and Web of Science) using the search terms: (Nigeria* OR “West Africa*” OR “Gulf of Guinea”) AND (“wood* fuel*” OR woodfuel* OR “wood-fuel*” OR “fuel wood” OR wood fuel OR “fuel-wood” OR “wood for fuel*” OR “fire wood” OR firewood OR “fire-wood” OR charcoal). Our usage of ‘wildcards’ varied according to database requirements. Records were exported on March 13th, 2023. After removing duplicates, this delivered 1,813 unique records. Title-, abstract- and full-text-screening then applied predetermined inclusion/exclusion criteria (see below). Subsets of 100 titles and 50 abstracts were screened by two independent reviewers. A Kappa score ≥ 0.6 was required to ensure consistent application of these criteria, although we consistently achieved scores > 0.9, indicating near-perfect agreement^81^.

We included literature in English, and available in full-text form (either open access or via the co-authors’ academic libraries). We required that an investigation included at least one empirical element, using primary data from one or more locations in Nigeria to assess either the severity of a given impact and/or the viability/success of a specific solution. We excluded studies using models or scenarios to project hypothetical future outcomes, studies using laboratory trials to compare the performance of different fuel/stove types, and remote sensing studies that did not apply ground-truthing to empirically link [land-use] change to wood fuel harvesting or an associated solution. We excluded papers that primarily examined the social or demographic determinants of behaviours (e.g. choice of cooking method or use of resources), as these typically focused on correlations rather than assessing the outcomes of specific interventions. However, causation was not a strict inclusion criterion. Studies were included if they reported observed, perceived, or anticipated impacts of wood fuel-related solutions, irrespective of whether causation was explicitly demonstrated. Many studies relied on associations, inferred impacts using proxy measures, or presented qualitative findings based on stakeholder perspectives rather than direct measurements that would have supported causation. Given this diversity in study designs and approaches, the findings reported in this paper should be interpreted as indicative rather than definitive causal relationships. The evidence strength associated with solution-related outcomes varied, depending largely on the methods used for data collection. Our approach to dealing with this is described below.

Research into solutions to wood fuel-related issues can vary widely. From our perspective, an ‘ideal’ investigation empirically measures change in the severity of one or more impacts following the implementation of a specific solution, while quantifying the various co-benefits, trade-offs and barriers experienced during or after implementation. However, preliminary searches suggested that such studies are relatively rare, and investigations more typically (a) quantify the extent of a given impact, and then prescribe possible solutions; (b) assess the success of an implemented solution using a proxy metric (e.g. uptake of an alternative cooking option) to infer change in the severity of an impact; or (c) examine people’s actions or experiences through questionnaires, interviews or discussion groups. In each case, co-benefits, trade-offs and barriers may (or may not) be identified or anticipated to varying degrees, so such studies were retained for further investigation. The reviewed literature included a mix of solution-oriented and target-oriented studies. While some explicitly assessed interventions and their outcomes (solution-oriented), others focused on broader SDG targets without directly evaluating specific interventions (target-oriented). Both study types were considered relevant to understanding the interactions between interventions and SDG-related impacts.

Information compiled from documents deemed eligible for inclusion included any references to wood fuel-related impacts and/or solutions (provided they were directly linked to one or more impacts) were recorded, along with any mentioned co-benefits, trade-offs or barriers. Information on these topics was first extracted in raw text form, and later grouped into logical categories. For each impact recorded, we noted the end-use of the fuel (if stated), as well as the metric with which it was quantified. For each solution, we recorded whether it had been implemented or was only suggested in the literature. Where solutions had been implemented, we recorded the metric (and changes therein) used to gauge effectiveness. Where solutions had not been implemented, any anticipated impacts were noted as expected outcomes rather than empirical findings. For each co-benefit, trade-off or barrier, we recorded whether it was observed directly, perceived or anticipated, but did not consider temporal aspects (e.g. that some trade-offs may reduce over time, or that some co-benefits may emerge with more time).

We did not assess the quality or risk of bias of the studies selected for our review. However, we assessed the evidence strength associated with a given solution-related outcome in a clearly defined method (see below) using a workshop to bring together empirical evidence and stakeholder knowledge in a way that is consistent with evidence-informed practice^82^.

### Stakeholder consultations

We convened a one-day workshop in Abuja, Nigeria in November 2023. The workshop involved 21 people with expertise in sectors spanning energy, environment, public health and sustainable development in Nigeria. Participant institutions included the Federal Ministries of Health and Environment (including Departments of Forestry and of Desertification, land Degradation and Drought Management), the National Agency for the Great Green Wall, the National Council on Climate Change, the Nigeria Governors Forum, the Renewable Energy Association of Nigeria, the NGO Agro-climatic Resilience in Semi-Arid landscapes, the Global Environmental and Climate Conservation Initiative, and Atmosfair Climate & Sustainability Limited. Working in groups, participants were provided with the lists of impacts and solutions identified through our REA but were not shown any other elements of our findings. They were asked to link individual solutions to specific impacts that they felt they could help address, or which might be aggravated as an unintended consequence. Participants were also asked to consider any barriers that could be faced when implementing each solution, along with any trade-offs or co-benefits (wood fuel-related or otherwise) that could potentially arise. Information was recorded by facilitators and consolidated with findings from the REA post-workshop. Participants were given the opportunity to suggest impacts or solutions that may not have been captured through the REA, but no further additions were made.

### Data consolidation and assessment of evidence strength

Recorded impacts and solutions were grouped into logical categories developed through discussions among co-authors, based on information gathered through our REA, and were subsequently used as the basis for eliciting information on barriers, co-benefits, and trade-offs at the stakeholder workshop. Recorded barriers, co-benefits and trade-offs were also grouped into logical categories in the same manner, using collated information from both sources.

Based on the above categories, we constructed matrices combining (1) all recorded combinations of impacts and the solutions observed/expected to mitigate them, and (2) combinations of solutions and the barriers that have been observed or might be expected to hinder their success (see Results and discussion section). By highlighting cases where a given single solution can or cannot address specific combinations of impacts, the impacts-solutions matrix also indicates, respectively, trade-offs and co-benefits directly pertaining to wood fuel impacts, for each solution. Trade-offs and co-benefits not pertaining to wood fuel impacts were more heterogeneous than other aspects of our enquiry, so are presented in narrative form, rather than as a matrix.

The strength of evidence associated with a given solution-related outcome depends on whether or not it has been directly observed, inferred using some proxy measure (e.g. a reduction in the quantity of wood consumed to infer a reduction in forest loss), or anticipated. Observed outcomes were those directly measured following solution implementation. Inferred outcomes relied on correlation-based or proxy indicators. Anticipated outcomes were those expected by study authors or stakeholders but should not be interpreted as causal evidence. To account for this, we present our findings (in colour-coded matrices) using a scheme consisting of four categories each with an increasing degree evidence strength: Anticipated outcomes with low associated evidence strength (“Anticipated (low)”) are where an outcome was anticipated *either* in the literature *or* by workshop attendees, while anticipated outcomes with high associated evidence strength (“Anticipated (high)”) are where an outcome was anticipated by both. Observed outcomes with low associated evidence strength (“Observed (low)”) are where direct observations have been made, either using a proxy measure to infer an outcome, or where comparisons were made between two existing contexts (e.g. wood fuel vs. non-wood fuel users), but with no counterfactual. Observed outcomes with high associated evidence strength (“Observed (high)”) are where outcomes have been directly observed following the implementation of a given solution.

## Data Availability

Data compiled for this work are available from the lead author, Jamie A. Carr, upon request.
